# Efficacy of low- and moderate-intensity statins for achieving low- density lipoprotein cholesterol targets in Thai type 2 diabetic patients

**DOI:** 10.1186/s40200-017-0290-x

**Published:** 2017-02-13

**Authors:** Nuntakorn Thongtang, Chaiyut Sitthananun, Sutin Sriussadaporn, Wannee Nitiyanant

**Affiliations:** Division of Endocrinology and Metabolism, Faculty of Medicine Siriraj Hospital, Mahidol University, 2 Wanglang Road, Bangkoknoi, Bangkok, 10700 Thailand

**Keywords:** Statins, Intensity, Diabetes, Asians

## Abstract

**Background:**

Low dose statins are commonly used among Asians, because plasma low-density lipoprotein cholesterol (LDL-C) reductions similar to those observed in Westerners are achieved at lower doses. We aimed to assess the efficacy of low- and moderate-intensity statins for achieving plasma lipid targets in Thai type2 diabetes (T2D) and to evaluate factors associated with greater LDL-C reduction by statins.

**Methods:**

T2D patients who were treated with low- and moderate-intensity statins at the Siriraj Diabetes Clinic during the January 2013 to December 2014 study period were eligible for inclusion(n = 978), 400 patients were randomly recruited. Patients were classified into 1 of the following 2 groups according to their plasma LDL-C reductions by statins (N = 393); very favorable response (LDL-C reduction ≥50%) or less favorable response (LDL-C reduction <50%).

**Results:**

Of the 400 patients, 41.3% were low-intensity statin users. Mean age was 64.4 ± 12.7 years, 64% were female. Median duration of diabetes was 13.3 years and mean HbA1C was 8.1 ± 1.9%. Plasma LDL-C goal of <100 mg/dl and <70 mg/dl was achieved in 84.3% and 38.0% respectively, with no significant difference between the low- and moderate-intensity statin users. LDL-C reductions ≥50% can be achieved in 38.4%. Factors associated with very favorable responses from statins were age, hypertension, patients with stable or reduced weight, and better glycemic control.

**Conclusion:**

Low- and moderate-intensity statins achieved plasma LDL-C goal of <100 mg/dl and <70 mg/dl in 84.3%, and 38.4% of the patients respectively. Due to the improved response to lower doses observed in Asians, a titration dosage strategy should be considered.

**Electronic supplementary material:**

The online version of this article (doi:10.1186/s40200-017-0290-x) contains supplementary material, which is available to authorized users.

## Background

Type 2 diabetes (T2D) is an independent risk factor for atherosclerotic cardiovascular disease (ASCVD) and has been classified as coronary heart disease (CHD) risk equivalent [1–3]. Statin treatment has been shown to be effective in reducing ASCVD events both in primary and secondary prevention [4, 5]. The American Diabetes Association 2016 guideline [6] and the American College of Cardiology/American Heart Association 2013 guideline [7] both recommended using moderate- and high-intensity statins in all T2D patients, except those aged <40 years without ASCVD risk factors. Low-, moderate-, and high-intensity statins therapy are predicted to reduce plasma low-density lipoprotein cholesterol (LDL-C) levels from baseline by approximately <30%, 30% to <50%, and ≥50%, respectively [7]. Low-intensity statins are not recommended in these guidelines for patients with T2D [6, 7]. However, previous data suggested that Asians achieved plasma LDL-C reductions that were similar to those observed in Westerners, except at lower statin doses [8–11]. A proposed explanation for this dose-effect difference was genetic variability in drug metabolism [12]. Treatments with simvastatin 10 mg/day and atorvastatin 10 mg/day over 8 weeks at 6 Asian centers resulted in average plasma LDL-C reductions of 35% and 43%, respectively [10]. In addition, blood statin levels were reported to be higher in Asians than in Westerners at the same statin doses, which suggest an increased risk of statin toxicity in Asian population. Lee, et al. reported a difference in plasma LDL-C reduction response to rosuvastatin between subjects of European and Asian ancestry living in Singapore [13]. Moreover, Asian subjects had approximately twofold greater plasma exposure to rosuvastatin than Caucasians, which was not the result of body weight or environmental factors [13]. Although high-intensity statin therapy has been used effectively and safely in Asian patients especially ASCVD patients, most statin side effects are dose-dependent [14–16]. Titration of statin dosage is a common approach among Thai physicians thus low- and moderate-intensity statins are commonly prescribed among Thai T2D patients without ASCVD.

The aim of this study was to assess the efficacy of low- and moderate-intensity statins for achieving plasma lipid targets in Thai T2D patients, and to assess factors associated with greater plasma LDL-C reductions during statin therapy.

## Methods

This retrospective cohort study was conducted at Siriraj hospital, Mahidol University– Thailand’s largest university-based national tertiary referral center. All of the study subjects are Asians. T2D patients who were treated with low- and moderate-intensity statins at the Siriraj Diabetes Clinic during the January 2013 to December 2014 study period were eligible for inclusion. Patients were stratified into 3 groups by statin intensity according to American College of Cardiology/American Heart Association (ACC/AHA) 2013 guideline; as low-, moderate-, or high-intensity (Additional file 1). Patients were included if they met the following criteria during the study period: (I) age ≥18 years; (II) diagnosed with T2D; (III) received low- to moderate-intensity stable statin doses for at least 2 months; and, (IV) had baseline lipid profile laboratory investigation. The medical records of all subjects were reviewed and demographic and clinical data, including diabetic complications, medications used, and blood chemistry, were recorded. The presence of ASCVD was defined as the presence of CHD, ischemic stroke, peripheral artery disease or aortic aneurysm diagnosed during the data collection. The protocol for this study was approved by the Siriraj Institutional Review Board (SIRB) ethical committee. This research was done in accordance with the Ethical Principles of Medical Research Involving Human Subject*s* outlined in the declaration of Helsinki in 1975.

**Fig. 1:**
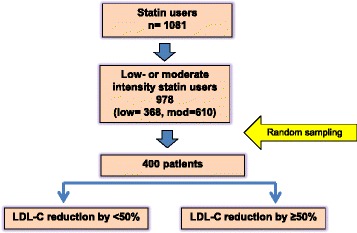
The Study Flow Chart

A total of 1,081 patients attended Siriraj Diabetes Clinic during January 2013 to December 2014 and were statin users. Low- and moderate-intensity statins were prescribed in 978 (90.47%) of the patients while high-intensity statins were prescribed in only 103 patents (9.53%). Of the 978 low- to moderate-intensity statin users and based on the results of the sample size formula shown below, 400 patients were randomly selected by using random sample numbers generating from an Excel program (Fig. 1). Baseline plasma LDL-C level before statin therapy was available in 393 patients. Percentages of plasma LDL-C reduction by statin therapy were calculated using the following formula:

$$ \left(\frac{\left(\mathrm{Pre}\hbox{-} \mathrm{stain}\;\mathrm{plasma}\;\mathrm{LDL}\hbox{-} \mathrm{C}\;\mathrm{level}\hbox{-} \mathrm{plasma}\;\mathrm{LDL}\hbox{-} \mathrm{C}\;\mathrm{at}\;\mathrm{the}\;\mathrm{time}\;\mathrm{of}\;\mathrm{enrollment}\right)}{\mathrm{Pre}\hbox{-} \mathrm{statin}\;\mathrm{plasma}\;\mathrm{LDL}\hbox{-} \mathrm{C}}\right)\;\mathrm{X}\;100 $$

Patients were classified into 1 of the following 2 groups according to their percentage plasma LDL-C reduction after statin treatments; very favorable (LDL-C reduction ≥50%) or less favorable response (LDL-C reduction <50%). Factors associated with greater plasma LDL-C reduction were analyzed (Fig. 1).

### Sample size calculations

We estimated that low-and moderate-intensity statins would achieve plasma LDL-C of <70 mg/dl in 35% of the subjects based on our own previous study (unpublished). A sample size of 350 subjects was required to obtain a 95% confidence interval with a margin error of 5% according to the formula: n = Z_α_^2^ P(1 – P)/d^2^. A sample size was round up to 400 subjects for approximately 14% sample loss.

Factors associated with greater plasma LDL-C reductions were calculated based on the formula using in the case control study. If baseline LDL ≥160 mg/dl is prevalent 43% and 23%, respectively in the group with LDL-C reduction ≥50% and <50%. A sample size of 124 per group is required for a ratio of case to control of 1:1 with a 95% confidence and a power of 90%.

In order to achieve both objectives, the sample size of 400 is used in this study.

### Statistical analysis

All statistical analyses were performed using SPSS software version 20 (SPSS, Inc, Chicago, IL, USA). Categorical variables were compared using Fisher’s exact test or chi-square analysis and continuous variables were compared using Student’s *t*-test or Mann–Whitney *U* test. Data are presented as numbers (%), percentages, mean ± SD, or median. All statistical tests were 2-tailed. A *p* value ≤0.05 was considered to be statistically significant.

## Results

### Demographic data

Of 400 patients, 165 patients (41%) used low-intensity statins and 235 patients (59%) used moderate-intensity statins. Patient demographic and clinical data are summarized in Table 1. Mean age of patients was 64.4 ± 12.7 years, and 64% were female. Study participants had a long duration of diabetes, with a median duration of 13.3 years. Mean HbA1C was 8.1 ± 1.9%. In most patients, statin drugs had been prescribed for an extended period of time. Median duration of statin use was 63 months. Patients who received moderate-intensity statins had significantly (*p* < 0.05) higher prevalence of ASCVD, CHD, chronic kidney disease (CKD), and higher systolic blood pressure (SBP) than patients who received low-intensity statins. Other clinical characteristics were similar between the low and moderate groups. Chronic diabetic complications were also not significantly different between groups. Pre-statin plasma lipid levels are showed in Table 1. Mean total cholesterol and mean plasma LDL-C were 219.3 ± 45.6 mg/dl and 138.9 ± 37.6 mg/dl, respectively. Patients who received moderate-intensity statins had significantly higher baseline (pre-statin treatment) plasma cholesterol, triglyceride, and LDL-C levels than patients in the low-intensity statin group. Pre-statin plasma HDL-C levels were also significantly lower in moderate-intensity statin users than in low-intensity statin users.

**Table 1:** Clinical Characteristics of the Study Participants

Characteristic	Overall (*n* =400)	Low-intensity statins (*n* = 165)	Moderate-intensity statins (*n* = 235)	*p-*value
Female	256(64.0%)	113(68.5%)	143(60.8%)	0.12
Age (mean ± SD, years)	64.4 ± 12.7	64.0 ± 13.5	64.7 ± 12.1	0.62
Duration of DM (median, years)	13	14	12	0.13
Duration of statins (median, months)^a^	63	63	61.5	0.46
Smoking	10/355(2.8%)	3/146 (2.1%)	7/209 (3.3%)	0.63
Drink alcohol	28/352 (8.0%)	7/146 (4.8%)	21/206 (10.2%)	0.06
Family history of CHD	5/186 (2.7%)	3/83 (3.6%)	2/103 (1.9%)	0.81
Weight before statin treatment (kg)	66.6 ± 13.1	66.8 + 13.4	66.4 + 12.9	0.74
Body weight at enrollment (kg)	67.9 ± 14.4	66.9 ± 12.5	68.5 ± 15.5	0.30
Body mass index (kg/m^2^)	26.4 ± 5.7	26.4 ± 4.8	26.4 ± 6.2	0.96
Waist circumference (cm)	93.5 ± 11.5	93.0 ± 11.7	93.9 ± 11.42	0.72
Systolic blood pressure (mmHg)	132.7 ± 14.8	130.7 ± 15.2	134.1 ± 14.4	0.02
Diastolic blood pressure (mmHg)	74.5 ± 11.0	73.47 ± 11.2	75.2 ± 10.9	0.13
Comorbidity				
ASCVD	79/400 (19.8%)	24/165 (14.5%)	55/235 (23.4%)	*0.03*
CHD	57/400 (14.3%)	16/165 (9.7%)	41/235 (17.4%)	*0.02*
Stroke	27/400 (6.8%)	9/165 (5.5%)	18/235 (7.7%)	0.39
Peripheral artery disease	11/400 (2.8%)	3/165 (1.8%)	8/235 (3.4%)	0.52
Aortic aneurysm	2/400 (0.5%)	0/165 (0.0%)	2/235 (0.9%)	0.64
Hypertension	296/400 (74.0%)	124/165 (75.2%)	172/235 (73.2%)	0.66
Chronic kidney disease	106/400 (26.5%)	34/165 (20.6%)	72/235 (30.6%)	*0.03*
Diabetic retinopathy				0.88
No DR	232/347 (66.9%)	102/140 (72.9%)	130/207 (62.8%)	
NPDR	83/347 (23.9%)	25/140 (17.9%)	58/207 (28.0%)	
PDR	32/347 (9.2%)	13/140 (9.3%)	19/207 (9.2%)	
Diabetic neuropathy				0.66
Normal	120/165 (72.7%)	45/65 (69.2%)	75/100 (75.0%)	
Impaired monofilament test	30/165 (18.2%)	14/65 (21.5%)	16/100 (16.0%)	
Foot ulcer	15/165 (9.1%)	6/65 (9.2%)	9/100 (9.0%)	
Diabetic nephropathy				0.43
MAU < 30 mg/g creatinine	175/296 (59.1%)	73/118 (61.9%)	102/178 (57.3%)	
MAU ≥ 30 mg/g creatinine	121/296 (40.9%)	45/118 (38.1%)	76/178 (42.7%)	
Pre-statin plasma lipid levels				
Total cholesterol (mg/dl)	219.3 ± 45.6	211.9 ± 40.3	224.6 ± 48.4	*0.01*
Triglyceride (mg/dl)	157.3 ± 78.7	143.1 ± 67.8	167.2 ± 84.3	*0.01*
HDL (mg/dl)	51.0 ± 15.1	53.0 ± 15.5	49.5 ± 14.8	*0.04*
LDL-C (mg/dl)	138.9 ± 37.6	132 ± 33.6	143.7 ± 39.6	*<0.01*

Data presented as number (%), mean ± SD, or median; *p*-value < 0.05 indicates statistical significance. ^a^Duration of statin was counted from the time of statin initiation until the data collection date. Pre-statin plasma LDL-C levels were available in 393 patients

CHD, coronary heart disease; DM, diabetes mellitus; BMI, body mass index; ASCVD, atherosclerotic cardiovascular disease; DR, diabetic retinopathy; NPDR, non-proliferative diabetic retinopathy; PDR, proliferative diabetic retinopathy; MAU, microalbuminuria;. HDL-C, high-density lipoprotein cholesterol; LDL-C, low-density lipoprotein cholesterol

### LDL-C goal achievement

**Fig. 2:**
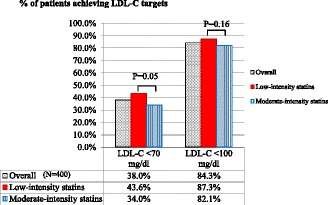
Plasma LDL-C Goal Achievement in Patients Treated with Low- or Moderate-intensity Statins

We found that 337 patients (84.3%) who received low- or moderate-intensity statins can achieve plasma LDL-C goal of less than 100 mg/dl, and 152 patients (38%) achieved LDL-C goal of less than 70 mg/dl (Fig. 2). Interestingly, moderate-intensity statins did not result in a significant greater LDL-C reduction goals achievement rate than the low-intensity statins. In fact, low-intensity statin users demonstrated a trend toward greater achievement of LDL-C reduction <70 mg/dl than moderate-intensity statin users. The percentages of patients achieving plasma LDL-C goals in the low- and moderate-intensity statin groups were 87.3% *vs.* 82.1%, respectively for plasma LDL-C goal of <100 mg/dl, and 43.6% *vs.* 34.0%, respectively for LDL-C goal <70 mg/dl (Fig. 2).

### Percentages of plasma LDL-C reductions by statins

**Fig. 3:**
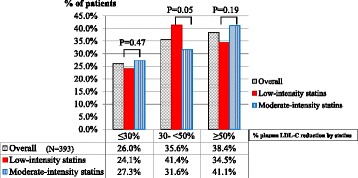
Percentage of Plasma LDL-C Reduction by Low- and Moderate-intensity Statin Therapy

The median percentage of plasma LDL-C reduction by statins of the overall participants (N = 393) were −34.6%, while low- and moderate-intensity statin group had median percentages of plasma LDL-C reduction by −31.4% and −36.1%, respectively. Moreover; we found that 75.9% of low-intensity statin users had plasma LDL-C reduction greater than 30% from baseline, which is higher than the LDL-C reduction expected in the Westerners. Moreover 41.1% of moderate-intensity statin users, and 34.5% of the low-intensity statin users had plasma LDL-C reduction ≥50% from baseline which is expected to achieved from high-intensity statins in the Westerners (Fig. 3). As compared to low-intensity statins, moderate-intensity statins did not result in a greater proportion of patients having percentage baseline LDL-C reduction of ≥50% or 30% to <50% than low-intensity statins (Fig. 3).

### Factors predicting greater plasma LDL-C reduction with statins

There were 38.4% (n = 151) of subjects who had plasma LDL-C reduction ≥50% from baseline and this group was defined as the very favorable response group. The remaining participants who had plasma LDL-C reduction <50% were classified as the less favorable response group (n = 242). Patients in the very favorable response group were significantly older and had longer duration of statin use than the lessfavorable response group. Hypertension was more prevalent in the very favorable response group. Moreover, the very favorable response group had a significantly greater number of patients who could maintain or reduce their body weight after statin therapy was initiated, as compared to the lessfavorable response group (Table 2). Blood chemistry results before statin treatment of both groups are shown in Table 2. Subjects with better glycemic control had greater LDL-C lowering response with low- and moderate-intensity statins than subjects with poorer glycemic controls. Mean baseline HbA1C were significantly lower in the very favorable response group than the less favorable response group (7.7% *vs.* 8.3%, *p* < 0.01). The very favorable response group had higher mean plasma total cholesterol and LDL-C levels than the less favorable response group (Table 2).

**Table 2:** Demographic and Clinical Characteristics of Patients by LDL-C Reduction Group (<50% *vs.* ≥50%)

	LDL-C reduction <50% (*n* = 242)	LDL-C reduction ≥50% (*n* = 151)	*p*-value
Female	152 (62.8%)	99 (65.6%)	0.58
Age (years)	63.2 ± 13	66.4 ± 12.1	*0.02*
Duration of DM (mo, median)	13	13	0.29
Duration of statin usage (mo, median)	56	88	*<0.01*
Smoking	7/215 (3.3%)	3/133 (2.3%)	0.86
Alcohol	17/213 (8.0%)	10/132 (7.6%)	0.89
Family history of CHD	1/112 (0.9%)	4/72 (5.6%)	0.08
Body mass index (kg/m^2^)	26.2 ± 4.9	26.6 ± 6.8	0.58
Weight before statin treatment (kg)	65.9 ± 12.6	67.4 ± 13.9	0.28
Waist circumference (cm)	94.6 ± 11.9	91.2 ± 10.4	0.19
Weight reduction	87/227 (38.3%)	68/138 (49.3%)	*0.04*
SBP (mmHg)	131.8 ± 14.3	74.9 ± 11.3	0.11
DBP (mmHg)	74.9 ± 11.3	73.8 ± 10.8	0.33
Comorbidities			
CHD	35/242 (14.5%)	20/151 (13.2%)	0.74
Stroke	18/242 (7.4%)	9/151 (6.0%)	0.57
Peripheral artery disease	6/242 (2.5%)	5/151 (3.3%)	0.63
Aortic aneurysm	2/242 (0.8%)	0/151 (0.0%)	0.26
Hypertension	167/242 (69.0%)	124/151 (82.0%)	*<0.01*
Chronic kidney disease	65/242 (26.9%)	40/151 (26.5%)	0.94
Diabetic retinopathy			0.79
Normal	135/206 (65.5%)	94/136 (69.1%)	
NPDR	51/206 (24.8%)	30/136 (22.1%)	
PDR	20/206 (9.7%)	12/136 (8.8%)	
Diabetic neuropathy			0.75
Normal	75/105 (71.4%)	43/58 (74.1%)	
Impaired Monofilament	21/105 (20.0%)	9/58 (15.5%)	
Foot ulcer	9/105 (8.6%)	6/58 (10.4%)	
Diabetic nephropathy			0.93
MAU ≥ 30 mg/g creatinine	71/175 (40.6%)	48/117 (41.0%)	
MAU < 30 mg/g creatinine	104/175 (59.4%)	69/117 (59.0%)	
**Statin potency**			0.19
Low-intensity statin users	106/242 (43.8%)	56/151 (37.1%)	
Moderate-intensity statin users	136/242 (56.2%)	95/151 (62.9%)	
Pre-statin blood chemistry			
Total cholesterol (mg/dl)	204.7 ± 43.8	242.4 ± 39.1	*<0.01*
Triglyceride (mg/dl)	153.5 ± 81.0	161.8 ± 74.6	0.35
HDL (mg/dl)	50.9 ± 16.3	51.1 ± 13.1	0.95
LDL-C (mg/dl)	125.7 ± 34.1	160.0 ± 33.2	*<0.01*
HbA1C (%)	8.3 ± 2.0	7.7 ± 1.6	*<0.01*
FBS (mg/dl, median)	158.9	169.4	0.32

Data presented as mean ± SD or median; *p*-value < 0.05 indicates statistical significance

*CHD*, coronary heart disease; *DM*, diabetes mellitus; *BMI*, body mass index; *ASCVD*, atherosclerotic cardiovascular disease; *DR*, diabetic retinopathy; *NPDR*, non-proliferative diabetic retinopathy; *PDR*, proliferative diabetic retinopathy; *MAU*, microalbuminuria; LDL-C, low-density lipoprotein cholesterol; SD, standard deviation; HDL-C, high-density lipoprotein cholesterol; FBS, fasting blood sugar

## Discussion

Low- and moderate-intensity statins were commonly used in Thai T2D patients, and were prescribed in 34.04% and 56.43%, respectively among statin users while high-intensity statins were prescribed in only 9.53%. We found that low- and moderate-intensity statins can achieve plasma LDL-C reduction goal of <100 and <70 mg/dl in 84.3% and 38% of the patients, respectively. Moreover low- and moderate-intensity statins resulted in plasma LDL-C reduction ≥50% in 34.5% and 41.1% of patients, respectively. In fact, plasma LDL reductions greater than 50% from baseline are expected to result from high-intensity statin therapy in the Westerners [7, 15]. Overall our data demonstrated greater efficacy of plasma LDL-C reductions with low doses of statins in Thai diabetic patients, which is consistent with the results of other studies in Japanese [9], Korean [8], and Chinese [10, 17] subjects. Matsuzawa Y*,* et al. found that 5 mg of simvastatin in Asians obtained the same LDL-C reduction effect as 20 mg of simvastatin use in Westerners [9]. A possible explanation for the greater effect of statin at lower doses may center on differences in statin pharmacokinetics and pharmacogenetics between Asian and Western populations resulted in higher blood statin levels in Asians than Westerners with the same statin dosage [11–13, 17, 18].

Interestingly, moderate-intensity statins do not produce greater LDL-C goal achievement rate or greater percentage of baseline LDL-C reduction, as compared with low-intensity statins in Thai diabetic patients. Doubling of statin dosage resulted in greater plasma LDL-C reduction of approximately 6% [19, 20], thus may explain the non-significant increase in LDL-C goal achieving rate in our study. However, a meta-analysis of statin trials reported greater CAD risk reduction with high-intensity statins as compared to the lower intensity statins [4]. It is still unclear whether this additional benefit is due to the greater LDL-C reduction or from other pleiotropic effects of statins. Moderate-potency statins were more commonly used than low-potency statins in our clinic, thus moderate-potency statin users were randomly recruited more into our study. The presence of ASCVD, the presence of cardiovascular risk factors such as hypertension, chronic kidney disease, and higher pre-statin plasma lipid levels were associated with physicians’ choice of using higher potency statins. These are reasonable approach according to the recent guidelines [6, 7].

Our study found that factors associated with greater plasma LDL-C reduction in response to low- and moderate-intensity statins were older age, longer duration of statin usage, hypertension, weight maintenance or weight loss during statin therapy, lower HbA1c, and higher baseline plasma lipids. Some of these factors were consistent with the previous studies. Con, et al. found that the probability of attaining the LDL-C goal increases with age [21]. Trompe, et al. studied characteristics of non-responders and high responders of pravastatin treatment among the elderly and found that patients who did not respond well to pravastatin therapy were, on average, 1 year younger [22]. Higher blood statin level in the elderly might be another explanation for the greater plasma LDL-C reduction response. The finding of longer duration of statin usages in the very favorable LDL-C reduction response group may also reflect the longer duration of hypercholesterolemia in the older patients. In addition, the ability to maintain body weight or lose weight predicts a better LDL-C lowering response during statin treatment. We speculate that the ability to maintain body weight or lose weight results from an improved self-perception of one’s health status. These patients may have healthier lifestyles and may engage in regular exercise in addition to statin treatment, so these patients may have better response to statin therapy. Moreover, a better glycemic control was another predictor of better LDL-C lowering response during statin therapy. This also supports the concept of better self-care in this group.

### Limitation

This study has some limitations. First, this was a retrospective study and plasma lipid levels were recorded only at baseline before statins initiation and at the last two visits. As such, biochemical changes between times and between changes in medications were not investigated. Second, we only evaluated low- and moderate-intensity statins as they were most commonly prescribed in Thailand for primary prevention of ASCVD; therefore, our data cannot be applied to high-intensity statins. Third, only 19.8% of our study population had ASCVD. As such, our findings can be applied mainly to T2D for primary prevention, but cannot be generalized for use in secondary prevention. Moreover, in a real-world setting, inter-individual variability in statin responsiveness may be more evident [23, 24].

## Conclusions

Thai diabetic patients primarily receive low- or moderate-intensity statins. Low- and moderate-intensity statins achieved the plasma LDL-C reduction goal of <100 and <70 mg/dl in 84.3% and 38% of the patients, respectively. Predictors of better response to statin therapy were older age, stable or reduced weight, and better glycemic control. Due to the improved response to lower doses observed in Asians, a titration dosage strategy should be considered.

## Additional file

Additional file 1: Statin intensity classification according to American College of Cardiology/American Heart Association (ACC/AHA) 2013 guideline. (PPTX 63 kb)

## Abbreviations

ASCVD: Atherosclerotic cardiovascular disease
CKD: Chronic kidney disease
CHD: Coronary heart disease
LDL-C: Low-density lipoprotein cholesterol
SBP: Systolic blood pressure
T2D: Type2 diabetes
