# Supplementary material for: Efficacy of low- and moderate-intensity statins for achieving low- density lipoprotein cholesterol targets in Thai type 2 diabetic patients
Source: J Diabetes Metab Disord. 2017 Feb 13;16:6. doi: 10.1186/s40200-017-0290-x (PMC5307884; doi:10.1186/s40200-017-0290-x)
Supplement: Additional file 1: — Statin intensity classification according to American College of Cardiology/American Heart Association (ACC/AHA) 2013 guideline. (PPTX 63 kb) [file 40200_2017_290_MOESM1_ESM.pptx]

## Slide 1
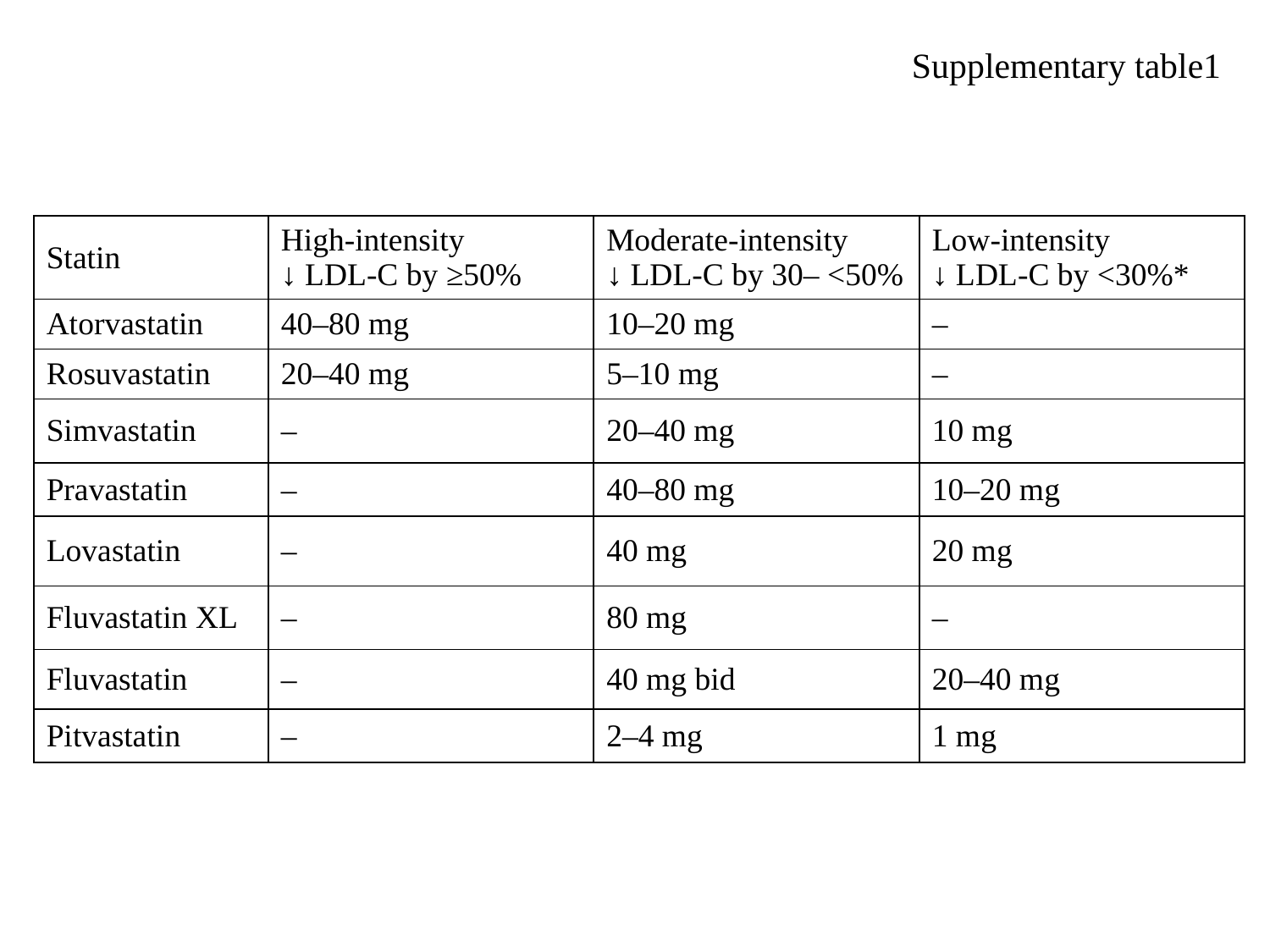

Supplementary table1
| Statin | High-intensity ↓ LDL-C by ≥50% | Moderate-intensity ↓ LDL-C by 30– <50% | Low-intensity ↓ LDL-C by <30%\* |
| --- | --- | --- | --- |
| Atorvastatin | 40–80 mg | 10–20 mg | – |
| Rosuvastatin | 20–40 mg | 5–10 mg | – |
| Simvastatin | – | 20–40 mg | 10 mg |
| Pravastatin | – | 40–80 mg | 10–20 mg |
| Lovastatin | – | 40 mg | 20 mg |
| Fluvastatin XL | – | 80 mg | – |
| Fluvastatin | – | 40 mg bid | 20–40 mg |
| Pitvastatin | – | 2–4 mg | 1 mg |
